# Developing social marketed individual preconception care consultations: Which consumer preferences should it meet?

**DOI:** 10.1111/hex.12555

**Published:** 2017-04-25

**Authors:** Sabine F. van Voorst, Chantal A. ten Kate, Lieke C. de Jong‐Potjer, Eric A. P. Steegers, Semiha Denktaş

**Affiliations:** ^1^ Department of Obstetrics and Gynecology Erasmus Medical Center Rotterdam The Netherlands; ^2^ Department of Social and Behavioural Sciences Erasmus University College Rotterdam The Netherlands

**Keywords:** health promotion, perinatal health, preconception care, reproductive health, social marketing

## Abstract

**Aims:**

Preconception care (PCC) is care that aims to improve the health of offspring by addressing risk factors in the pre‐pregnancy period. Consultations are recognized as a method to promote perinatal health. However, prospective parents underutilize PCC services. Uptake can improve if delivery approaches satisfy consumer preferences. Aim of this study was to identify preferences of women (consumers) as a first step to social marketed individual PCC consultations.

**Methods:**

In depth, semi‐structured interviews were performed to identify women's views regarding the four components of the social marketing model: product (individual PCC consultation), place (setting), promotion (how women are made aware of the product) and price (costs). Participants were recruited from general practices and a midwife's practice. Content analysis was performed by systematic coding with NVIVO software.

**Results:**

The 39 participants reflected a multiethnic intermediately educated population. *Product:* Many participants had little knowledge of the need and the benefits of the product. Regarding the content of PCC, they wish to address fertility concerns and social aspects of parenthood. PCC was seen as an informing and coaching service with a predominant role for health‐care professionals. *Place:* the general practitioner and midwife setting was the most mentioned setting. *Promotion:* A professional led promotion approach was preferred. *Price:* Introduction of a fee for PCC consultations will make people reconsider their need for a consultation and could exclude vulnerable patients from utilization.

**Conclusion:**

This study provides consumer orientated data to design a social marketed delivery approach for individual PCC consultations.

## 1 INTRODUCTION

Preconception care (PCC) includes all measures taken before conception to increase the health of the prospective mother (parents) and child. It addresses risks associated with adverse pregnancy outcomes. The large number of acknowledged preconceptional risk factors can be categorized into 13 domains: health promotion, immunizations, infectious diseases, chronic medical conditions, psychiatric conditions, maternal exposures, genetic risks, medication, nutrition, environmental risks, psychosocial stressors, reproductive history and special groups.^1^, ^2^ Whilst some risks and interventions are applicable to all couples (e.g. lifestyle recommendations, folic acid supplementation), some risks are only present amongst some individuals (e.g. a positive family history for hereditary diseases).

Preconception care has been acknowledged as a valuable addition to perinatal health care, to improve and reduce inequalities in perinatal health and women's health.^3^, ^4^ Many countries are facing challenges regarding which approach for the delivery of PCC is best suited to their health‐care setting. In the Netherlands, the Dutch Health Council advocates PCC for the general public in the form of individual consultations.^5^ Rationale is that the majority of couples in the general population is known to have at least one risk factor for which PCC would be useful.^6^ Furthermore, a consultation with a health‐care professional provides the opportunity for individual risk assessment and intervention. However, despite availability of tools and guidelines, PCC consultations are only offered at a small scale.^7^ When offered, uptake is low due to hesitancy amongst people to utilize PCC.^8^, ^9^ In order to increase the utilization of individual PCC consultations, we need to address the question of how this service should be delivered in order to meet demands and preferences of prospective parents. Using a consumer‐oriented approach to change behaviour of a target group (namely uptake of PCC services) is the basis of social marketing. Social marketing is defined as “the application of commercial marketing technologies to the analysis, planning, execution and evaluation of programmes designed to influence voluntary adoption of recommended behaviours by a targeted audience in order to improve their personal welfare and that of society.”^10^ One of the steps is applying a marketing mix in which “product,” “price,” “place” and “promotion” characteristics are blended in a marketing plan that reflects the appropriate mix of these four “P”s. The right “*product”* has to be backed by the right “*promotion”* and put in the right “*place”* at the right “*price.”* 11

Social marketing has been suggested to develop approaches for the delivery of preconception care.^12^, ^13^ As the Dutch health system advocates delivery of PCC in the form of individual PCC consultations, this study is confined to the “*product”* of individual PCC consultations. Goal of the product is primarily to promote a healthy pregnancy and to reduce the chances of adverse pregnancy outcomes. A consultation constitutes a thorough risk assessment to identify risks that warrant intervention or counselling in the preconception phase. “*Promotion”* concentrates on the promotion of individual preconception care. The 3rd P, “*place,”* addresses characteristics of the setting. The 4th P, “*price,”* includes the costs for patients for this product.

Aim of this study was to identify consumers’ preferences regarding these marketing components as a first step in designing a socially marketed delivery approach for individual preconception care.

## 2 METHODS

This study is a prospective, community based, qualitative study.

### 2.1 Study population

Participants were enrolled via purposive sampling from waiting rooms at two general practices (GP) and one midwife practice participating in the Healthy Pregnancy 4 All study.^14^ Staff of the practice asked women whether they would allow for a medical student to explain a study in which they could participate. These women attended their respective practices for a scheduled appointment for other health issues. If women were open to talk about participation in a study, a medical student (CtK) explained the study and assessed the participants’ eligibility. Women in the reproductive age range (18‐42 years) who did not exclude a future pregnancy were eligible (see Appendix S1 for script). Insufficient proficiency of the Dutch or English language was defined as an exclusion criterion. If women agreed to participate, they filled in a questionnaire on baseline characteristics and the interview was scheduled at a convenient time at the respective practice. Sample size was set at 40 interviews. Fewer interviews were deemed sufficient if theoretical saturation would be reached at an earlier point.

### 2.2 Data collection

Data collection consisted of individual semi‐structured interviews. The topic list was designed to address each “P” of the marketing mix. Questions were formulated to identify aspects of the 4′ ps which authors had brainstormed to be important and which are known to be of importance in literature. As the interviews proceeded, the interview strategy was adapted slightly, to ensure that participants understood the questions. The topic list contained 27 open‐ended questions—with scripted subquestions when relevant—(Appendix S1). To ensure successful discussion about individual PCC, we provided a definition of our product: individual PCC consultations. All interviews were recorded and transcribed verbatim. Participants filled in a questionnaire on baseline characteristics. The ethics committee approved the study (MEC 2013‐586). All participants provided informed consent for the recording and the use of data.

### 2.3 Data analysis

The interview transcripts were analysed to identify elements of the social marketing model. Analyses were performed with NVIVO software for qualitative analysis of data.^15^ After the data were imported, a basic coding scheme was made according to each P of the social marketing model. This coding scheme was piloted. Two researchers independently applied the coding scheme to 10 interviews and discussed discrepancies and modification of the nodes to optimally fit the content of the interviews. This led to a definitive codebook. The remainder of the interviews was coded by one researcher and checked by the other researcher. With the matrix coding function and query function of NVIVO, contents could be analysed to identify contents (perceptions of respondents) and patterns in contents (consistency, frequency). Quotes were extracted to illustrate findings. The quotes were translated from Dutch to English (by a native speaker) and back again (by a second translator) to verify consistency of the translation.

## 3 RESULTS

### 3.1 Study population

Forty women were recruited. One interview was discontinued because the candidate did not speak Dutch or English sufficiently to understand and answer the questions. After the 36th interview, no new information was provided and it was decided to stop data collection after 39 interviews. Twenty‐three participants were recruited from the midwifery setting, and 16 participants were recruited from the GP setting. Mean interview time was 22 minutes. Table 1 presents the characteristics of the study participants. Participants were between 21 and 38 years old and reflect a multiethnic, intermediately educated population. At the time of the interview, 56% of the participants were pregnant. Most non‐pregnant participants did not contemplate pregnancy within the next 6 months.

**Table 1:** Characteristics of participants

		N (%) Total = 39
Age	Median age (years)	27.97 (21‐38)
Obstetric history	Nulliparous	19 (48.7)
	Multiparous	20 (51.3)
Maternity	Zero children	25 (64.1)
	One child	10 (25.6)
	Two children	3 (7.7)
	Three children	1 (2.6)
Current pregnancy wish	Pregnant at the moment	22 (56.4)
	Planning pregnancy <3 months	1 (2.6)
	Planning pregnancy 3‐6 months	0
	Planning pregnancy >6 months	16 (41.0)
Marital status	Married	22 (56.4)
	Cohabiting	9 (23.1)
	In a non‐cohabiting relationship	5 (12.8)
	Single	3 (7.7)
Ethnicity^a^	Dutch	26 (66.7)
	Surinamese	2 (5.1)
	Turkish	1 (2.6)
	Moroccan	3 (7.7
	Other	7 (17.9)
Educational attainment level^b^	Low	4 (10.3)
	Intermediate	17 (43.7)
	High	16 (41.0)
	Other	2 (5.0)

Numbers reflect number of participants (N) unless specified differently.

a Ethnicity is defined as the social or cultural group that the participant considered themselves to be part of.

b Educational attainment level was classified according to the International Standard Classification of Education (ISCED).^33^

### 3.2 Consumer preferences for individual PCC consultations

**Figure 1:**
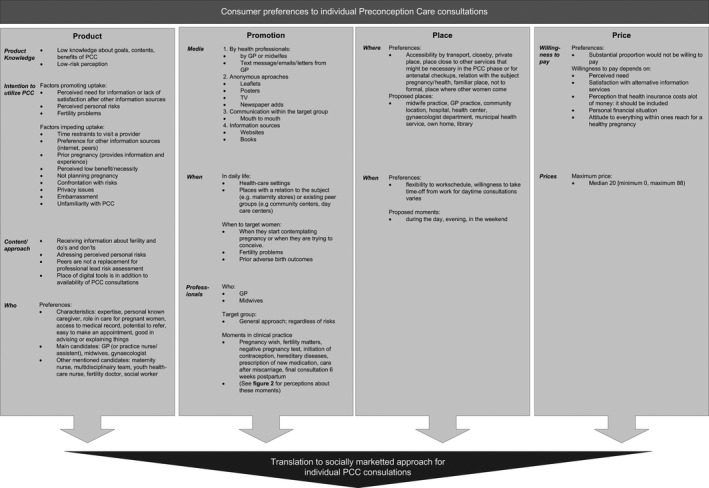
Perceptions and preferences of women regarding the four components of social marketing model: Product, Promotion, Place and Price. Items are listed according to the frequency they were mentioned

Figure**** 1 displays the preferences of women within each construct of the social marketing model. We will describe and illustrate the most important findings in this section.

#### 3.2.1 Product

##### Knowledge of the product

Knowledge about the purpose and the contents of PCC consultations was low amongst participants. The majority presumed PCC to be fertility‐related care. Its aim was “*to help women get pregnant,”* hastened by many participants with “*as fast as possible”* or “*within the desired time frame.”* Participants also thought its goal would be to help women with decisions about parenthood. In line with these presumed goals, participants mentioned that the target group would consist of women in the pre‐pregnancy period ranging from women considering having a child to subfertile women. In line with this, presumed content would be education about fertility and diagnostic work‐up and treatment of subfertility.

##### Utilization

After participants were informed about what PCC actually was, intentions to utilize PCC varied. Reported reasons to utilize PCC were mostly to be informed on their questions about perceived risks and fertility. Participants reported they would be more likely to utilize PCC consultations after trying to become pregnant for a longer period or when they are becoming pregnant for the first time. Multiparity was reported as a reason not to utilize PCC, because most participants thought they would know enough after prior pregnancy experiences. Lack of perceived need or benefit of a PCC consultation was the most recurrent theme, as one respondent said illustratively*:*

I still believe my body protects the fetus against harmful exposures during the first three months. Secondly, it has been going fine without the existence of PCC services in the past, so it will be fine regardless.

Practical considerations (e.g. having to take time‐off from work), having other information sources or feelings about interference in the privacy and spontaneity of conception were other reasons not to utilize PCC consultations.

##### Preferred contents and approaches

Regarding the contents of PCC, participants preferred PCC to address fertility, questions about their perceived risks and about parenthood. In line with this, the most mentioned approach for the consultation was the provision of information. A few participants mentioned a preference for a coaching approach:

You can stop with contraception; however it would be better if you were coached in the course of becoming pregnant instead of – ‘well ok I'm just going to stop with contraceptives, and just see what happens.’

Although contact with peers and the use of tools (apps, internet, questionnaires) were valued positively, participants valued them as an addition to professional lead PCC consultations rather than a replacement. The personal approach, the authority and the credibility of a health‐care professional were the most important advantages of a PCC consultation by a professional. Participants mentioned the lack of credibility of the information and privacy issues, as the main disadvantage of forementioned tools. A hallmark for tools with trustworthy information sources and a function in tools where questions could be placed for answering by a health‐care professional were suggested improvements.

##### Providers

Expertise, trust and involvement in care for pregnant women were mentioned as the most important prerequisites of PCC providers. Based on these attributes GP, midwives and gynaecologists were most frequently suggested as PCC providers. Delegation of care to a nurse/nurse practitioner/medical assistant within general practices was deemed appropriate.

#### 3.2.2 Promotion

**Figure 2:**
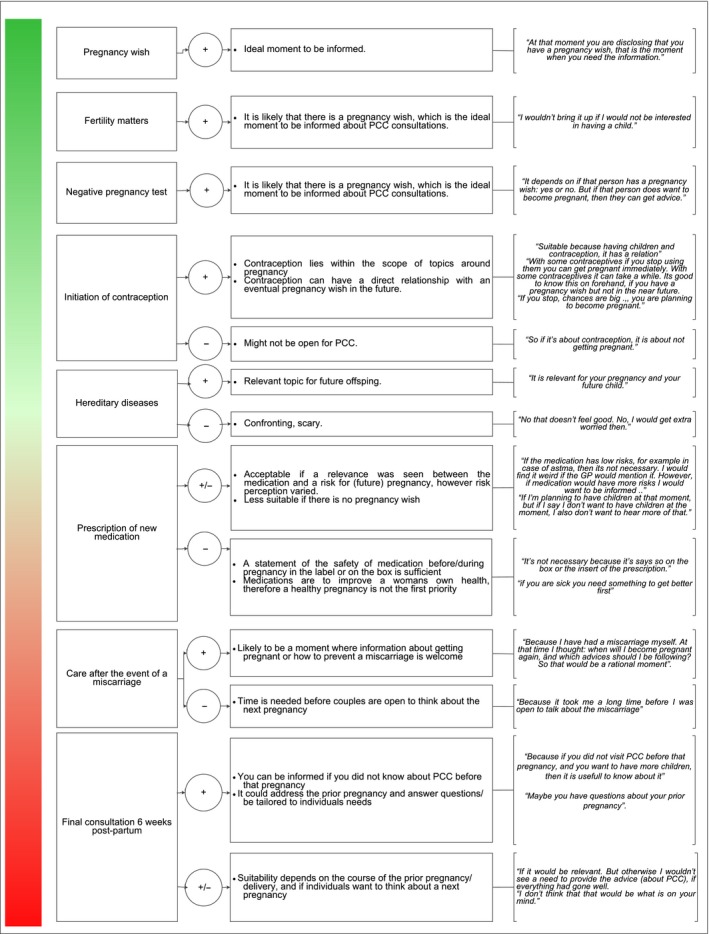
Suitability of moments for health‐care providers to promote a preconception care consultation—according to participants. Participants were asked to rate (grades 1‐ 10) the suitability of moments in routine care for a health provider to point out the opportunity to have a PCC consultation. Based on these grades, moments were ranked from being most suitable (top, green) to being least suitable (bottom, red)

Four communication approaches to make women aware of PCC were mentioned by participants (see Figure 1). The most preferred way to be informed about PCC was through a professional, mostly directly or indirectly via an email, text message or a letter. GP were seen as the most suitable professionals to do so as they are the starting point for health care in the Dutch Health system, and everybody has a GP. Midwives were also seen as suitable professionals to promote PCC. However, participants mentioned that people generally associate midwives to care during pregnancy. Figure 2 displays perceptions about the suitability of contact moments with GP and midwives to be informed about PCC.

In the promotion of PCC, participants preferred a general approach in which professionals promote PCC to all women so everybody would be enabled to make an informed decision whether or not they would utilize PCC. Suitable places for the promotion of PCC were all related to either pregnancy or the target group. Participants preferred to be made aware of PCC when they start thinking about becoming pregnant or when they are trying to become pregnant. They mentioned that this is most likely when they have a stable life, being married or having finished education. There is understanding that these factors differ per person and that early promotion of PCC is necessary to reach women in time. Participants realized that caregivers generally do not know whether women are planning a pregnancy or not.

#### 3.2.3 Place

Accessibility, in terms of distance and convenience with public transport, was the most important prerequisite. Other recurrent preferences of the location were privacy, location close to other services related to PCC (e.g. access to midwifery care or dietician if needed), familiar places or places where other women would come. These attributes caused participants to mention primary care places (midwifery practices, GP, health centres) or hospitals (where gynaecologist/ specialist care takes place) as suitable settings for a PCC consultation. At home, municipal health centres and community centres were also mentioned.

Flexibility to consumers’ working schedules was the most mentioned prerequisite regarding time. With differences in willingness to take time‐off from work between participants, consultations in the evening or even in the weekend were mentioned to be preferred or even essential to some.

#### 3.2.4 Price

Willingness to pay is mostly related to own financial situations and perceptions about reimbursement of health care in the Dutch system—where health insurance is mandatory and perceived as expensive. The requirement to pay for PCC would make a substantial proportion of participants seek other (free) alternatives for a PCC consultation or to re‐evaluate their need for a PCC consultation. This could provide a dilemma, for instance to women on social benefits.

Just financially speaking, if it is not reimbursed, it would not be convenient, because I am on social welfare, I have fixed expenses, and sometimes at the end of the month it's difficult to pay them and I have to stick it out. My children are always my priority.

According to participants, PCC should be reimbursed because it is preventive care. If they had to pay, the majority would be willing to pay a fee below 25 euro.

## 4 DISCUSSION

### 4.1 Summary of findings

This manuscript presents consumer research to drive socially marketed strategies for delivery of individual PCC consultations. The most profound finding was the lack of knowledge about the content and potential benefits of the product. Fertility and psychosocial aspects of parenthood are components which should be added to PCC. This study points out a key role for health professionals to promote PCC during moments in routine care with an explainable link to relevance of PCC. Participants find the community‐based primary care setting (GP and midwives) to be the most suitable *place* for PCC. Regarding *price,* a fee will influence who is reached with the PCC service.

### 4.2 Comparison to the literature

This is not the first study to employ a social marketing approach within the field of PCC; however, studies define their product differently. Lewis and co‐authors define their product as preconception health and performed a formative inquiry regarding women's preferences regarding preconception health.^13^ Quinn and co‐authors confined their product to a single preconception measure: preconception folic acid supplements. Their intervention approach was a collective campaign.^16^ To our knowledge, this is the first study in which the product is confined to a specific approach for PCC, namely individual comprehensive PCC consultations.

To our knowledge, there are no studies assessing the effectiveness of social marketing approaches for preconception care in terms of uptake of services or behavioural change.

Perceptions about preconception care have been assessed in numerous studies. Regarding the “*product,”* the general misconception of the need and perceived benefits of preparing for a healthy pregnancy has been acknowledged as the primary challenge to overcome in the delivery of preconception care.^6^, ^17^, ^18^, ^19^ The need to address fertility and psychosocial aspects of parenting during PCC is in line with reported low knowledge about fertility (e.g. fertile days) and timing of parenthood.^20^ Regarding “*place,”* prior studies underline the preference of women for GP and midwives to be the primary providers of PCC.^9^, ^17^, ^21^, ^22^ Regarding “*promotion,”* it has been recommended that health‐care professionals point out PCC in the event of a negative pregnancy test, when birth control is discussed and in the check‐up following delivery of a baby.^17^, ^23^, ^24^ This study supports that the proposed moments are in line with women's preferences. To our knowledge, there are no studies that assess the effectiveness of pointing out PCC during routine daily care in terms of promotion of the uptake of PCC.

### 4.3 Strengths and limitations

We believe one of the strengths of this study is that the product is confined to a specific approach: individual comprehensive preconception care. Firstly, findings over the remaining P's are valid as respondents are all talking about the same approach to PCC. Secondly, this way the social marketed intervention plan is in line with recommendations of the Dutch health board and guidelines of GP and midwives.^5^, ^25^, ^26^ By taking these points into account, results are close to the situation in practice, which is important for feasibility of implementation of the approaches which derive from our findings.

Ideally studies about PCC are performed with a study population that is trying to conceive. However, these women are not detectable within the general population. Therefore, we employed a second best approach: women were included if they did not exclude having a pregnancy wish in the future. This caused the study population to include women throughout various stages of their reproductive life. We believe our study population to be a representative study sample of planners and non‐planners and nulliparous and multiparous women. We explored patterns regarding planners/ non‐planners; nulliparous/ multiparous and women with prior adverse pregnancy outcomes. However, preferences regarding components of the social marketing plan were not consistent within these groups, due to the small size of these subgroups. A limitation due to the recruitment in GP and midwifery practices is that results only apply to women that utilize health care. We recommend effectiveness of approaches that derive from our findings to be evaluated for subgroups to fine‐tune intervention strategies.

This study presents findings in the Netherlands, where individual comprehensive PCC consultations in primary care are advocated in policy and guidelines. Many countries explore roles of GP and midwives in the delivery of PCC.^24^, ^27^, ^28^, ^29^, ^30^, ^31^, ^32^ Findings of this study could be valuable to such countries or countries with a strongly developed primary care system seeking an individual approach to preconception care. Furthermore, the methods of consumer research employed in this study could be illustrative to other countries with other preferred approaches to PCC.

## 5 CONCLUSIONS AND PRACTICAL IMPLICATIONS

Preferences of women are largely in line with how PCC is intended to be delivered by primary care givers. Explicit matters that need rethinking are (i) *product:* adding fertility matters and psychosocial aspects of parenthood to the contents of PCC, (ii) *place*: how PCC can be can be made accessible to subgroups such as the working population and the low health literate population and (iii) *price*: PCC is currently not reimbursed within basic health insurance whilst a substantial proportion of women is not willing to pay for individual PCC.

The most profound finding in this study was the low knowledge about contents, benefits and availability of individual PCC consultations. This emphasizes the importance of promotion. Participants point out a central role for GP and midwives in promoting PCC. They should feel empowered to promote PCC during the proposed moments. Furthermore, they should point out PCC regardless of the presence of risk factors; participants prefer to know about availability of PCC, so they can decide whether they want to utilize PCC. However, the low knowledge about PCC and the fact that midwives generally do not see non‐pregnant women provide rationale for a campaign within the public health sector additional to efforts of PCC providers. This would reach women that do not visit health‐care providers and it could sensitize the public to messages about PCC from health‐care providers

Our consumer research provides the foundation for a socially marketed programmatic approach to individual PCC care. An approach needs to be designed in which the identified preferences are met. The low knowledge and perceived need for PCC entails that there is a need for a continuous promotion strategy parallel to delivery of PCC. A promotion campaign needs to be developed and evaluated regarding their comprehensiveness and appeal to different target groups. Feasibility of meeting women's preferences needs to be evaluated with PCC providers and policymakers. The designed programme needs to be delivered iteratively, with continuous monitoring and adaption to specific target audiences.

## CONFLICTS OF INTEREST

No conflict of interests have been declared.

## AUTHORS’ CONTRIBUTIONS

SVV and CTK conceived the study design, coordinated, transcribed and coded the interviews, analysed data and drafted and revised the manuscript. LDJ and EAPS participated in study design and revision. SD participated in the study design and coordinated analysis and revision. All authors read and approved the final manuscript.
