# Supplementary material for: Developing social marketed individual preconception care consultations: Which consumer preferences should it meet?
Source: Health Expect. 2017 Apr 25;20(5):1106–13. doi: 10.1111/hex.12555 (PMC5600215; doi:10.1111/hex.12555)
Supplement: Supplementary file 1 [file HEX-20-1106-s001.docx]

**Appendix S1:** Semi-structured item list.

- Brief introduction to the study:

*“Purpose of this interview study is to learn how individual preconception care consultations could best be offered, according to women.”*

- Check if participant complies with inclusion criteria.

*“Therefore we ask women between 18 and 41 years old, that wish to have a child in the near future, later in life or are sure not to have a completed family yet, to participate. Does this apply to you?”*

- The participant has filled in the base-line questionnaire.

**Product**

**A. Preknowledge** Firstly assess what people know about the product.

1. What is a PCC consultation? (**Preknowledge)**
2. What do you think is the goal of PCC consultation? (**Preknowledge)**
3. Would you utilize the possibility of a PCC consultation if you had a child wish? (**Preknowledge)**
4. Why would or wouldn’t you make use of a PCC consultation? (**Preknowledge)**

**B. Presentation with our product**

Assure that from now on the interview is about the product.

- *I’ll now explain our vision on preconception care, so we are sure we are talking about the same thing for the rest of the interview. It is possible to visit a preconception care service if you are thinking about becoming pregnant. During the consultation you can ask questions about fertility, getting pregnant,* your *health and you will receive advice about what you can do to be optimally prepared for a healthy pregnancy. In the first months the baby is very vulnerable: for example important organs such as heart and lungs are formed. During the PCC consultation advice is given about your personal health (e.g. regarding medication use because they can give deformities to the fetus, or regarding hereditary diseases) and general advice is given (for example folic acid supplementation). Preconception care consultations are for everybody in any stage of pregnancy contemplation. Furthermore it includes a thorough risk analysis after which individual advice is provided.”*

1. After this explanation, would you utilize the possibility of a Preconception Care Consultation if you had a child wish? Why or why not? **(Utilization of product)**
2. What would be important reasons for you to utilize an individual preconception care consultation service? **(Utilization of product)**
3. What would be barriers to utilize an individual preconception care consultation service? **(Utilization of product)**
4. What would you like to address during a preconception care consultation regarding your pregnancy wish? (**Content of consultation)**?
5. Who should deliver individual PCC to you? **(Delivery by who)**
6. What makes this person the most appropriate provider of individual PCC to you? **(Delivery by who)**
7. Should individual PCC always be delivered by a health care professional? Yes/ no and why? **(Alternatives)**
8. Which alternatives for delivery by a health care professional would you find suitable? **(Alternatives)**
9. What is your opinion about digital media to provide you with preconception information (for example an app or website)? (**Alternatives)**
10. Could this replace an individual consultation with a health care professional? **(Alternatives)**

**Promotion**

1. How would you prefer to be informed about the possibility to visit a health care professional for a PCC consultation? **(How)**
2. Which moments in daily life would you find suitable to be informed about a PCC consultation? **(When)**
3. During which phase in your life would you like to be appointed the possibility to visit a health care professional for a PCC consultation? **(When)**
4. Which health care professional would you prefer to inform you about the opportunity to visit a health care professional for a PCC consultation (by this professional or by a different professional)? **(Who)**
5. During the next few questions I will mention moments during which you might visit a health care professionals. How suitable are these moments for health care professionals to point out the possibility of a preconception care consultation? Please tell us why moments are or aren’t suitable and provide a grade between 1 -10 for their suitability (1 being absolutely unsuitable and 10 being very suitable). **(When)**
6. When I mention my pregnancy wish
7. During regular follow-up of a chronic disease (e.g. check-up for Diabetes)
8. When hereditary diseases are discussed
9. When a medication is prescribed to me, which hasn’t been prescribed to me before
10. When contraception is discussed
11. When a pregnancy test is done and turns out to be negative
12. When I have questions about fertility
13. In the care after a miscarriage
14. During a consultation after I had a baby
15. When there are/ were health problems with my baby/child
16. a. What do you think about a more anonymous approach to inform you about the possibility of an individual PCC consultation?
    b. Would you prefer an anonymous approach or an approach by a professional or somebody else? **(How)**
17. Would you only prefer to be informed when you have risks or always (regardless of your health)? **(When)**

**Place**

1. What do you find important regarding the place where the individual PCC consultations are provided? **(Where)**
2. Where would you like the PCC consultation to be provided? **(Where)**
3. Which moment would you find suitable for a PCC consultation to take place? **(When)**
4. What is important regarding the moment at which PCC is offered? **(When)**

**Price**

1. Would you be willing to pay for a PCC consultation? Why or why not? **(Acceptance)**
2. How much would you maximally be willing to pay for a PCC consultation? **(Amount)**

- *Do you have any remaining remarks or questions?*

*We thank you for your participation.*
